# Acceptance of Electronic Labeling for Medicinal Product Information Among Malaysian Hospital Patients: Cross-Sectional Study

**DOI:** 10.2196/56591

**Published:** 2024-09-18

**Authors:** Xin Yee Loh, Ai Ling Woo, Azwa Haris, Cheryl Shajini Pereira, Bee Kim Tan

**Affiliations:** 1 School of Pharmacy Faculty of Health and Medical Sciences Taylor's University Subang Jaya Malaysia; 2 AstraZeneca Limited Petaling Jaya Malaysia; 3 Department of Pharmacy University Malaya Medical Centre Kuala Lumpur Malaysia; 4 Digital Health and Medical Advancement Impact Lab Subang Jaya Malaysia

**Keywords:** electronic labeling, e-labeling, electronic medication information, patient preference, acceptance, hospital ambulatory care patient

## Abstract

**Background:**

While perceptions of electronic labeling (e-labeling) in developed countries have been generally positive, existing data primarily come from studies involving hospital pharmacists, community pharmacy customers who may not be frequent medication users, and individuals receiving COVID-19 vaccines.

**Objective:**

This study aims to assess e-labeling acceptance, perceptions of its benefits, challenges with its implementation, and preferences among hospital ambulatory care patients in Malaysia. Additionally, the study investigates the factors influencing patients’ acceptance of e-labeling.

**Methods:**

A cross-sectional study using a 28-item questionnaire was conducted at the outpatient pharmacy department of a quaternary hospital in Kuala Lumpur, Malaysia, from May to June 2023. The questionnaire was developed based on a review of published literature related to e-labeling and was guided by the Unified Theory of Acceptance and Use of Technology, second version (UTAUT2). Patients aged 18 years and above were recruited using a stratified sampling method to ensure representative age-related medication usage. A mobile tablet was provided to patients for self-completion of the e-survey in their preferred language (English, Malay, or Mandarin). Categorical data on e-labeling acceptance, perceptions, and preferences were analyzed using descriptive statistics. Qualitative content analysis was performed to characterize participants’ responses to open-ended questions. Univariate and multivariate binomial logistic regression analyses were conducted to identify predictors of e-labeling acceptance.

**Results:**

Out of 462 patients approached, 387 (83.8%) participated in the survey, with 283 (73.1%) accepting e-labeling. Most participants perceived the electronic version of the package insert as beneficial, particularly for understanding their medication better through the choice of language (352/387, 91.0%). However, around half of the participants (197/387, 50.9%) expressed concerns about the potential risks of obtaining illegal medication information via e-labeling. Most participants (302/387, 78.0%) preferred to access electronic leaflets through government websites. However, 221/387 (57.1%) still wanted the option to request printed leaflets. Significant predictors of e-labeling acceptance included perceived benefits such as better understanding of medication (adjusted odds ratio [AOR] 8.02, 95% CI 2.80-22.97, *P*<.001), environmental protection (AOR 7.24, 95% CI 3.00-17.51, *P*<.001), and flexibility in information retrieval (AOR 2.66, 95% CI 1.11-6.35, *P*=.03). Conversely, being of Chinese ethnicity compared with Malay (AOR 0.28, 95% CI 0.13-0.60, *P*=.005) and perceived lack of self-efficacy in browsing electronic leaflets (AOR 0.25, 95% CI 0.11-0.56, *P*<.001) were associated with lower acceptance.

**Conclusions:**

The acceptance rate for e-labeling among hospital ambulatory care patients was moderately high and was significantly influenced by ethnicity as well as patients’ perceived benefits and challenges related to its implementation. Future strategies to enhance e-labeling uptake should address patient concerns regarding the challenges of using the digital platform and emphasize the benefits of e-labeling.

## Introduction

Pouliot et al [1] defined medication literacy as an individual’s ability to make safe decisions regarding medications and health based on the processing of patient-centered medication information (eg, written, oral, and visual). Patients with limited medication literacy often struggle with reading medication labels, understanding printed care instructions and health advice, and tend to use medications inappropriately. They are also less adherent to therapy [2,3]. The National Health Morbidity Survey Malaysia 2019 reported that 1 in 3 adult Malaysians has poor health literacy [4]. The high prevalence of poor health literacy among the public is concerning, as those with low health literacy are more likely to incur higher health care costs, placing a tremendous burden on the health care system. Despite health care professionals (HCPs) conveying medication information verbally, most patients have limited cognitive ability to retain orally transmitted information [5]. Therefore, medicinal product information leaflets can serve as a useful aid, in addition to verbal counseling, to address postconsultation gaps [6]. In Malaysia, there are 2 categories of health authority–approved medicinal product information: Package Inserts (PIs) and Consumer Medication Information Leaflets (RiMUP) [7]. RiMUP is written in layman’s terms and is available in both English and Malay. While PIs are legally required to be printed and enclosed with all products containing scheduled poisons and injectable over-the-counter medicines, distributing RiMUP with the product is optional.

Patients who receive and read medicinal product information leaflets are more likely to discuss their medications with their health care providers. This creates an opportunity for providers to use the leaflets as educational material to improve treatment knowledge and facilitate shared decision-making [8]. A majority (64.9%) of participants in a Saudi Arabian study reported that their medication adherence improved after reading medicinal product information leaflets [9]. However, 55% of respondents in a survey conducted by the European Association of Hospital Pharmacists revealed that hospital patients do not receive medicinal product information leaflets [10]. Furthermore, patients reported several issues with paper PIs that hinder their effective use, such as undersized fonts, medical jargon, and the lack of options in local languages [9,11,12].

Electronic labeling (e-labeling) is an emerging trend in disseminating legally approved medicinal product information in dynamic formats, such as XML. By leveraging digital advancements, e-labeling systems enable the use of personalized medication information to meet the specific needs of both patients and HCPs. In today’s digital age, as the public increasingly seeks online health information, e-labeling offers a convenient way to access regulatory-approved medicinal product information through a trusted channel [13]. E-labeling not only streamlines the updating process, enabling prompt dissemination of medicinal product information to a wide range of HCPs and patients, but also creates opportunities for integration into digital health services. This can support e-prescribing by reducing the risk of medication incompatibilities and enhancing patient safety. From the industry’s perspective, electronic provision of medicinal product information reduces logistical challenges in label updates, lowers printing costs for PIs, and improves the efficiency of the global pharmaceutical supply chain through shared labeling between countries. These advantages of e-labeling collectively contribute to achieving the United Nations Sustainable Development Goals (UNSDG) 3 (Good health and well-being) and 12 (Responsible consumption and production) [14].

To date, e-labeling is regulated at varying levels across different regions worldwide. The United States Food and Drug Administration (FDA) has mandated the electronic distribution of PIs since 2015 [15]. By contrast, only selected hospital medicinal products in the Baltic countries have been granted marketing authorization through e-labeling [12]. In Asia, Japan’s Pharmaceuticals and Medical Devices Agency (PMDA) officially enforced the removal of paper PIs for prescription drugs and medical devices in July 2023, aiming to transition toward a paperless system [16]. Malaysia’s National Pharmaceutical Regulatory Agency (NPRA) released the Guideline on Electronic Labelling (E-labeling) for Pharmaceutical Products, which came into effect on May 1, 2023. According to this guideline, approved PIs, RiMUPs, or both must be provided electronically via a machine-readable QR code on the product’s outer carton or inner label, linking to the NPRA QUEST system. The implementation of e-labeling is voluntary and applies to newly registered pharmaceutical products, biologics, and generic products containing scheduled poisons. An extension to other categories of products is still under review [17].

Understanding patients’ acceptance, perception, and preferences regarding e-labeling can help implement a more patient-centric approach to foster engagement. Such data are lacking in Malaysia, a developing country in Southeast Asia with a multiethnic population and a unique socioeconomic context that could influence the public’s readiness to adopt new digital health services. While perceptions of e-labeling in developed countries are generally positive, concerns have been reported, particularly among older adults and those with low digital literacy. Moreover, data representing patients were primarily obtained from HCPs [10], customers visiting community pharmacies who may not be frequent medication users [11], and individuals receiving COVID-19 vaccines [18]. These data cannot be generalized to hospital ambulatory care patients, who are primarily managing chronic diseases and require ongoing medication information for self-administration. Therefore, this study aimed to assess e-labeling acceptance, perceptions of its benefits, challenges with local implementation in Malaysia, and preferences among hospital ambulatory care patients. Additionally, the factors influencing patients’ acceptance of e-labeling were investigated.

## Methods

### Study Design and Population

A cross-sectional study was conducted at the University Malaya Medical Centre (UMMC), a quaternary teaching hospital with 1617 beds and multidisciplinary clinics spanning 40 clinical specialties. Established in 1962 and located in Kuala Lumpur, the capital of Malaysia, UMMC served as the study site. The study population consisted of a convenience sample of patients who visited the outpatient pharmacy department of UMMC. The inclusion criteria were patients aged 18 years and older; collecting prescription medications; and capable of reading English, Malay, or Mandarin. Exclusion criteria were patients with limited cognitive abilities, those collecting medication on behalf of others, those who were unwell, or those who refused to participate.

The calculated minimum sample size was 386, based on government hospital outpatient statistics from 2020, which totaled 16,635,350 [19]. This calculation used a 5% margin of error, a confidence level of 95%, and a response distribution of 50% [20]. A stratified sampling method was used based on the estimated proportions of prescription drug users: 18.0% under 40 years of age, 46.0% between 40 and 64 years of age, and 85.0% over 65 years old, to ensure age representativeness [21].

### Questionnaire

The study instrument was a 28-item questionnaire developed based on a review of the published literature related to e-labeling and informed by the Unified Theory of Acceptance and Use of Technology, second version (UTAUT2) [22]. The questionnaire consisted of 3 sections/pages: (A) demographics (4 items) and utility of medicinal product PIs (6 items), (B) perceptions of the benefits (6 items) and challenges with e-labeling implementation (5 items), and (C) acceptance (1 item) and preferences regarding e-labeling (6 items). Acceptance, as defined by Adell et al [23], is the willingness to use a system based on theoretical knowledge or experience.

The demographic information of the participants included age, gender, ethnicity, and education level. Age was categorized into 3 groups: 18-39, 40-64, and 65 and older. Ethnicity was categorized into Malay, Chinese, Indian, and other, while education level was classified as university/college, secondary school, primary school, and no formal schooling. The utility characteristics of medicinal product PIs captured included the sources of written medicine information, reasons for choosing these sources, and the frequency and reasons for reading PIs. Participants’ practices regarding the frequency of using PIs were rated as follows: always, sometimes, only when receiving new medication, or never. Acceptance, perceived benefits (such as ease of retrieval, medication understanding, personalization, up-to-date information, and environmental protection), perceived challenges (including issues with electronic gadgets, digital literacy, internet access, and label security), and preferences regarding e-labeling (such as format, access, and options) were assessed using a 5-point Likert scale, ranging from 1=strongly disagree to 5=strongly agree. The questions were presented in a choice format, except for 2 open-ended questions designed to elicit reasons for not reading PIs and to gather additional views from participants regarding e-labeling.

The content validity of the questionnaire was assessed by 6 subject matter experts using a 4-point Likert scale to evaluate the relevance of each survey item. The experts included 2 regulatory pharmacists, 2 hospital pharmacists, and 2 academic pharmacists. The degree of relevance was categorized into 2 groups: “not relevant” and “somewhat relevant” were considered as “0=irrelevant,” while “quite relevant” and “very relevant” were considered as “1=relevant.” The scale-level content validity index based on the average method and the universal agreement method were 0.97 and 0.85, respectively, meeting the satisfactory level (≥0.83). The wording of some questions and choices was modified following a discussion within the research team based on the feedback received.

The English version of the questionnaire was translated into Malay and Mandarin using forward and backward translation methods. The translation was performed by 2 native Malay speakers proficient in English. The translations were reviewed by the research team and reconciled into an optimal version based on the appropriateness of the wording. This reconciled version was then back-translated into English by 2 additional native Malay speakers with a strong command of English. The original English version of the questionnaire and its translations were compared by the research team, and any discrepancies were discussed (Multimedia Appendix 1). Revisions were made to the Malay version of the questionnaire as needed (Multimedia Appendix 2). A similar translation method was used for the Chinese version of the questionnaire (Multimedia Appendix 3).

To ensure the feasibility of the recruitment procedure and the face validity of the questionnaire, a pilot study was conducted with 30 participants (10 for each language: English, Malay, and Mandarin) through cognitive debriefing to assess clarity and understanding. Cronbach α coefficients were calculated, resulting in .73 for section B on perceived benefits and .79 for challenges with e-labeling implementation. The usability and technical functionality of the electronic questionnaire were tested before the pilot study.

### Data Collection

Data collection took place from May to June 2023 at the outpatient pharmacy department of UMMC. Potential participants were approached by XYL and invited to participate while waiting for their prescriptions to be filled. Those who agreed to participate were briefed on the study objectives and the estimated time required to complete the survey. A tablet was provided to each participant to indicate their informed consent, followed by the self-completion of the e-survey form in their preferred language (English, Malay, or Mandarin). The survey was designed to be open, allowing participants to review and change their answers using a back button. Completeness was ensured before survey submission through mandatory items in the e-survey form. Participation was voluntary, and participants could opt out without facing any negative consequences. No incentives were provided. Participants’ data were anonymized and stored in a password-protected file.

### Data Analysis

Statistical analysis was performed using the SPSS software (version 27; IBM Corp.). Descriptive statistics, including frequencies and percentages, were generated for all categorical variables. To facilitate analysis, responses for acceptance, perceived benefits, perceived challenges, and preferences were dichotomized into 2 categories: “strongly disagree,” “disagree,” and “neutral” were classified as “No,” while “agree” and “strongly agree” were classified as “Yes.” Univariate logistic regression was used to test the effect of each independent variable (demographic characteristics, utility of medicine PI, perceived benefits, and perceived challenges) on the probability of acceptance of e-labeling. Covariates with *P*<.25 were selected [24] and subsequently tested in a multivariate logistic regression model to identify significant predictors of e-labeling acceptance. *P* values <.05 in the multivariate logistic regression model were considered statistically significant.

A qualitative content analysis was performed following the 8 steps outlined by Zhang and Wildemuth [25] to characterize participants’ responses to the open-ended questions. The procedures included the following: (1) Importing participants’ response text data into qualitative data analysis software (NVivo version 10; Lumivero). (2) Coding data related to participants’ reasons for not reading the PI and their opinions on e-labeling implementation. (3) A coding scheme and a list of initial categories were developed using the constant comparison method. (4) To validate the coding scheme and ensure consistency, 2 researchers (XYL and BKT) independently coded the data from the first 5 participants. The coding by both researchers was found to be in agreement. (5) XYL then coded the remaining data and added new categories as needed. (6) BKT assessed the coding consistency against the raw data. (7) The categories/themes were refined based on the patterns observed in the coded data. Homogeneity of codes within each category and heterogeneity of codes across categories were reviewed to ensure there was no overlap; and finally, (8) the inductive content analysis process and results were reported descriptively.

### Ethics Approval

The study was granted ethics approval by the Medical Ethics Committee of UMMC (MREC ID Number: 2023214-12138, dated April 3, 2023), conducted in accordance with the Declaration of Helsinki, and reported according to the CHERRIES (Checklist for Reporting Results of Internet E-Surveys) checklist [26].

## Results

### Participant Demographics and Characteristics of Package Insert Use

Out of the 462 patients approached, 387 agreed to participate and completed the e-survey, resulting in a response rate of 83.8%. Participant demographics and characteristics of PI use are summarized in Tables 1 and 2. Participants were predominantly male (n=202, 52.2%), Chinese (n=185, 47.8%), aged 40-64 years (n=173, 44.7%), and had a university/college education (n=289, 74.7%). Among the 387 participants, more than three-quarters (n=312, 80.6%) reported seeking written information about their medication. PI was the second most popular source of written medicine information, with a utility rate of 34.6% (n=108), following the internet (n=188, 60.3%). By contrast, only 1.6% (n=5) of participants read the RiMUP published on the NPRA website. The internet was perceived as more readily accessible (162/299, 54.2%, vs 76/176, 43.2%) and easier to understand (98/299, 32.8%, vs 48/176, 27.3%) compared with PI, but was considered less trustworthy (16/299, 5.4%, vs 47/176, 26.7%). Most participants read the PI only when they received a new medication (n=125, 40.1%).

Among the 48 responses to an open-ended question, reasons for not reading the PI included the leaflets being voluminous and containing too much information (n=15, 31.2%), small font size that is hard to read (n=10, 20.8%), difficulty understanding medical terms (n=9, 18.7%), preference for Google due to convenience (n=6, 12.5%), the paper being too small (n=5, 10.4%), and already being well-informed by doctors (n=3, 6.2%). Conversely, side effects (228/824, 27.7%) and information on the medication’s purpose and how it works (224/824, 27.2%) were the main reasons for reading the PI (Tables 1 and 2).

**Table 1:** Participant demographic (N=387) and characteristics of package insert use (Ns=312).

Demographic characteristics and package insert utility			Values, n (%)		
**Age (years)**					
	18-39	108 (27.9)			
	40-64	173 (44.7)			
	65 and above	106 (27.4)			
**Gender**					
	Male	202 (52.2)			
	Female	185 (47.8)			
**Ethnicity**					
	Malay	109 (28.2)			
	Chinese	185 (47.8)			
	Indian and others	93 (24.0)			
**Highest level of education**					
	University/college	289 (74.7)			
	Secondary school	92 (23.8)			
	Primary school	5 (1.3)			
	No formal schooling	1 (0.3)			
**Obtain or seek medicine written information**					
	Yes	312 (80.6)			
	No	75 (19.4)			
**Frequency of package insert use**					
	Only when I receive a new medication	125 (40.1)			
	Sometimes	119 (38.1)			
	Always	40 (12.8)			
	Never	28 (9.0)			
**Reason for package insert use (n=824)**					
	Side effects	228 (27.7)			
	Medication purpose and how it works	224 (27.2)			
	Dosage or administration	190 (23.1)			
	Drug interactions or precaution with other diseases	143 (17.4)			
	Safety in pregnancy and breastfeeding	34 (4.1)			
	Others	5 (0.6)			

**Table 2:** Source of written information about medicine (N=312).

Source of written information about medicine	Total, n (%)	Reasons for the chosen source^a^, n/N (%)				
		Trustworthy	Easy to understand	Readily accessible	Recommended by others	Other reasons
Internet (eg, Google)	188 (60.3)	16/299 (5.4)	98/299 (32.8)	162/299 (54.2)	11/299 (3.7)	12/299 (4.0)
Package insert	108 (34.6)	47/176 (26.7)	48/176 (27.3)	76/176 (43.2)	4/176 (2.3)	1/176 (0.6)
Leaflets from health care professionals	8 (2.6)	4/12 (33.3)	7/12 (58.3)	1/12 (8.3)	0/12 (0)	0/12 (0)
RiMUP^b^ on the NPRA^c^ website	5 (1.6)	2/8 (25.0)	2/8 (25.0)	4/8 (50.0)	0/8 (0)	0/8 (0)
Others	3 (1.0)	3/7 (42.9)	1/7 (14.3)	2/7 (28.6)	1/7 (14.3)	0/7 (0)

^a^Participants can choose more than 1 reason for the chosen source of written information about medicine.

^b^RiMUP: Consumer Medication Information Leaflets.

^c^NPRA: National Pharmaceutical Regulatory Agency.

### Perceived Benefits and Challenges With e-Labeling Implementation

**Figure 1:**
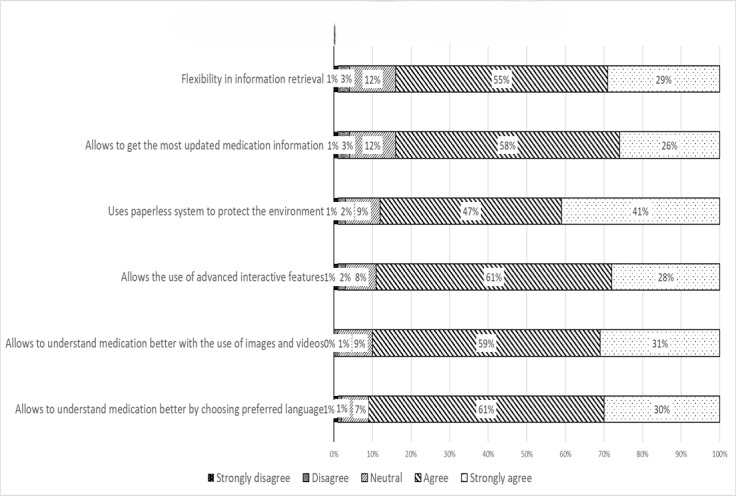
Participants’ perceived benefits toward electronic labeling implementation.

Most participants strongly agreed or agreed that the electronic version of the PI is beneficial, with 352/387 (91.0%) appreciating the ability to understand their medication better through their preferred language, 348/387 (89.9%) valuing the inclusion of images and videos, and 344/387 (88.9%) benefiting from advanced features such as adjustable font size and keyword search. Participants also agreed that e-labeling could help protect the environment by reducing paper use (340/387, 87.9%); provide the most up-to-date medication information (325/387, 84.0%); and allow access to information anywhere, anytime, without the fear of losing it (325/387, 84.0%; Figure 1).

**Figure 2:**
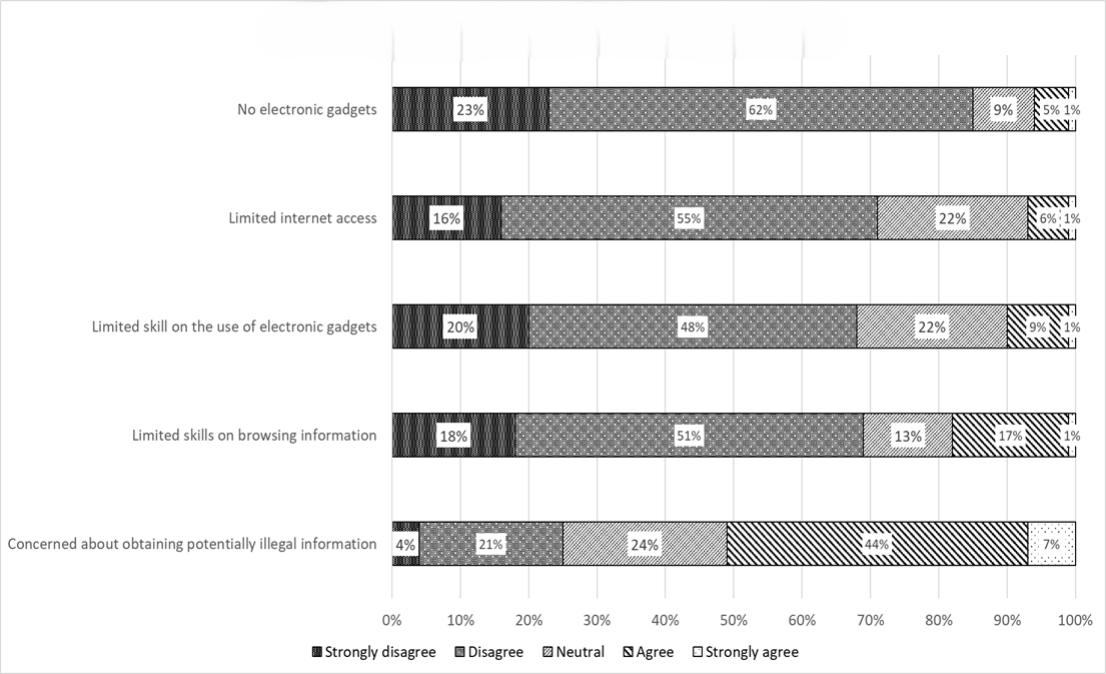
Participants’ perceived challenge towards electronic labeling implementation.

At the same time, around half of the participants (197/387, 50.9%) were concerned about obtaining potentially illegal medication information via e-labeling. A minority of participants expressed concerns about limited skills in browsing electronic medicinal product information (70/387, 18.1%), limited skills in using electronic gadgets (39/387, 10.1%), limited internet access (27/387, 7.0%), and not owning electronic gadgets (23/387, 5.9%; Figure 2).

### Acceptance of e-Labeling and Influencing Factors

**Figure 3:**
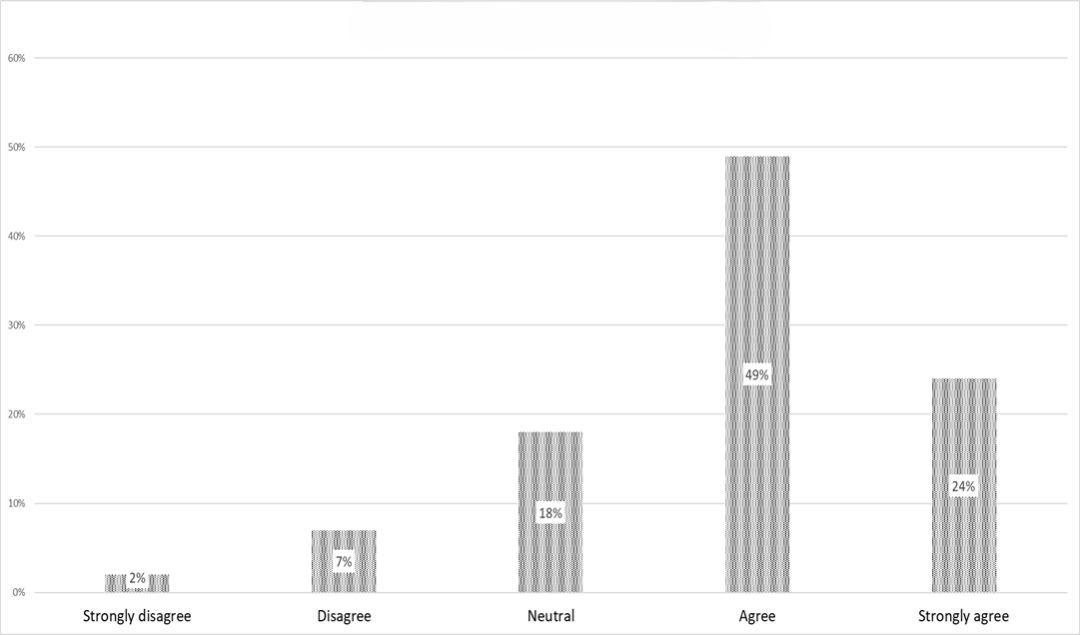
Participant’s acceptance of electronic labeling.

Overall, the participants’ acceptance rate of e-labeling was moderately high at 283/387 (73.1%; Figure 3). Univariate regression analysis revealed that all independent variables—including demographic characteristics, utility of PI, perceived benefits, and perceived challenges with e-labeling implementation—were potential factors associated with e-labeling acceptance (*P*<.25; Table 3).

Using a forward stepwise elimination method, multivariate regression analysis (Table 3) showed that participants who perceived a benefit in understanding medication better through images and videos were 8 times more likely to accept e-labeling (adjusted odds ratio [AOR] 8.02, 95% CI 2.80-22.97, *P*<.001). Those who perceived a benefit in using a paperless system to protect the environment had a 7 times higher probability of acceptance (AOR 7.24, 95% CI 3.00-17.51, *P*<.001) and those who perceived a benefit in being able to retrieve information anywhere, anytime, and without fear of losing it had 2 times the likelihood of accepting e-labeling (AOR 2.66, 95% CI 1.11-6.35, *P*=.03). By contrast, Chinese ethnicity was associated with a 72% lower probability of accepting e-labeling compared with Malay ethnicity (AOR 0.28, 95% CI 0.13-0.60, *P*=.005). Participants who perceived limited skills in browsing electronic medicinal product information were 75% less likely to accept e-labeling (AOR 0.25, 95% CI 0.11-0.56, *P*<.001). The binary logistic regression model was statistically significant (*χ*^2^_6_=49.285, *P*<.001). The model explained 39.3% of the variance in e-labeling acceptance (Nagelkerke *R*^2^). The Hosmer and Lemeshow test indicated that the model was a good fit for the data (*P*=.21, >0.05). Overall, the model had a good accuracy rate of 84% and exhibited excellent sensitivity (96.6%) in predicting e-labeling acceptance.

**Table 3:** Factors associated with participants’ acceptance of e-labeling.

Independent variables				Crude odds ratio (95% CI)		*P* value		Adjusted odds ratio (95% CI)		*P* value
**Demographics and characteristics of package insert use**										
	**Age (years)**									
		18-39	Reference		Reference		N/A^a^		N/A	
		40-64	0.62 (0.34-1.15)		.13^b^		N/A		N/A	
		65 and above	0.29 (0.16-0.56)		<.001^b^		N/A		N/A	
	**Gender**									
		Male	Reference		Reference		N/A		N/A	
		Female	0.66 (0.42-1.04)		.08^b^		N/A		N/A	
	**Ethnicity**									
		Malay	Reference		Reference		Reference		Reference	
		Chinese	0.38 (0.21-0.68)		.001^b^		0.28 (0.13-0.60)		.001^c^	
		Indian and others	0.88 (0.43-1.80)		.73		1.61 (0.58-4.51)		.36	
	**Highest level of education**									
		Secondary school and below	Reference		Reference		N/A		N/A	
		University/college	1.69 (1.03-2.78)		.04^b^		N/A		N/A	
	**Obtain or seek written information about medicine**									
		No	Reference		Reference		N/A		N/A	
		Yes	1.39 (0.80-2.40)		.24^b^		N/A		N/A	
	**Source of written information about medicine**									
		Other sources	Reference		Reference		N/A		N/A	
		Product inserts	0.71 (0.42-1.20)		.20^b^		N/A		N/A	
	**Frequency of reading the medicinal product package insert**									
		Never	Reference		Reference		N/A		N/A	
		Always	0.36 (0.11-1.16)		.09^b^		N/A		N/A	
		Sometimes	1.01 (0.35-2.98)		.98		N/A		N/A	
		Only when I received a new medication	0.50 (0.18-1.41)		.19^b^		N/A		N/A	
**Perceived benefits**										
	**Flexibility in information retrieval**									
		No	Reference		Reference		Reference		Reference	
		Yes	4.73 (2.70-8.26)		<.001^b^		2.66 (1.11-6.35)		.03^c^	
	**Allows to understand medication better with images and videos**									
		No	Reference		Reference		Reference		Reference	
		Yes	8.88 (4.32-18.25)		<.001^b^		8.02 (2.80-22.97)		<.001^c^	
	**Allows to understand medication better by choosing preferred language**									
		No	Reference		Reference		N/A		N/A	
		Yes	4.06 (1.98-8.33)		<.001^b^		N/A		N/A	
	**Allows the use of advanced interactive features**									
		No	Reference		Reference		N/A		N/A	
		Yes	2.99 (1.52-5.86)		.001^b^		N/A		N/A	
	**Allows to get the most updated medication information**									
		No	Reference		Reference		N/A		Reference	
		Yes	3.61 (2.04-6.41)		<.001^b^		N/A		N/A	
	**Uses a paperless system to protect the environment**									
		No	Reference		Reference		Reference		Reference	
		Yes	8.08 (4.15-15.74)		<.001^b^		7.24 (3.00-17.51)		<.001^c^	
**Perceived challenges**										
	**No electronic gadgets**									
		No	Reference		Reference		N/A		N/A	
		Yes	0.37 (0.16-0.87)		.02^b^		N/A		N/A	
	**Limited skill in the use of electronic gadgets**									
		No	Reference		Reference		N/A		N/A	
		Yes	0.40 (0.20-0.80)		.009^b^		N/A		N/A	
	**Limited internet access**									
		No	Reference		Reference		N/A		N/A	
		Yes	0.34 (0.15-0.77)		.01^b^		N/A		N/A	
	**Limited skills in browsing information**									
		No	Reference		Reference		Reference		Reference	
		Yes	0.25 (0.15-0.44)		<.001^b^		0.25 (0.11-0.56)		<.001^c^	
	**Concerned about obtaining potentially illegal information**									
		No	Reference		Reference		N/A		N/A	
		Yes	0.54 (0.34-0.85)		.008^b^		N/A		N/A	

^a^N/A: not applicable.

^b^*P*<.25.

^c^*P*<.05.

### Preference Toward e-Labeling

**Figure 4:**
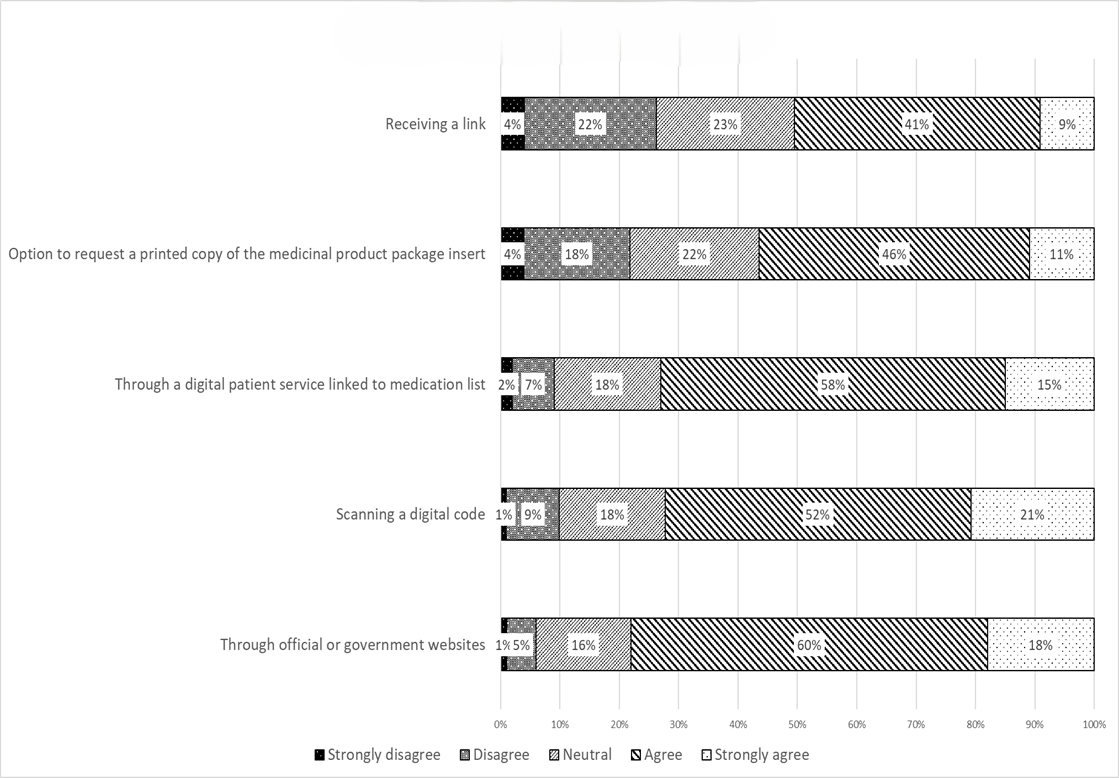
Participant’s preference toward electronic labeling.

Most participants preferred accessing electronic medicinal product information through official or government websites (302/387, 78.0%). Participants also showed interest in scanning a digital code, such as a QR code printed on the outer medication package (282/387, 72.9%), or accessing information through digital patient services, such as medication apps (282/387, 72.9%), compared with receiving a link via SMS text message or email (194/387, 50.1%). However, 221/387 (57.1%) of participants still preferred the option to request a printed copy of the medicinal product information (Figure 4).

In response to the open-ended question about views on e-labeling implementation for medicinal product information, most participants (33/83, 40%) emphasized that the e-labeling platform should consistently provide updated medication information that is neutral and free from product advertisements. They also highlighted the importance of the platform being easily accessible, user-friendly, easy to understand, and compatible with various electronic devices. Some participants also expressed that the content of the electronic label (e-label) must be reliable and protected from third-party modifications or cybersecurity attacks to ensure it is safe for patient use (7/83, 8%). Suggestions included accessing e-labeling through hospital websites verified by competent authorities. Additionally, participants recommended features for the e-labeling platform, such as the ability to compare information across medications for the same indication, separate sections for medication information on different diseases to facilitate easy location, links to journal or research articles, a section for user feedback, and a notification function to alert patients about new updates (6/83, 7%). As e-labeling for medicinal product information is a new initiative, participants also felt that a helpline should be available for patients needing assistance (3/83, 4%).

Despite the generally positive perception of e-labeling for medicinal product information, some participants expressed concerns about certain populations, including older adults, individuals with low digital literacy, those without internet access, and those without electronic devices (23/83, 28%). They suggested that it might be necessary to provide both paper and electronic inserts and recommended that authorities implement the e-labeling initiative in phases to allow the public time to adapt to the new platform (10/83, 12%).

## Discussion

### Principal Findings and Comparison With Prior Work

We found a moderately high acceptance rate (283/387, 73.1%) for e-labeling among hospital patients, with more than half (221/387, 57.1%) preferring to retain the option to request a printed copy. Most participants viewed the electronic version of the PI as beneficial, especially for understanding their medication better through language choices (352/387, 91.0%). However, around half of the participants (197/387, 50.9%) were concerned about the potential risk of accessing illegal medication information via e-labeling. Most participants preferred accessing e-labels from trusted sources such as government websites (302/387, 78.0%). Acceptance of e-labeling was significantly influenced by patients’ perceptions of benefits, including a better understanding of medication, environmental protection, and flexibility in information retrieval. By contrast, patients of Chinese ethnicity and those who perceived limited skills in using electronic inserts were less likely to accept e-labeling.

Compared with older studies, the acceptance rate for e-labeling in our study was higher. For example, a study conducted in Sweden before the pandemic reported that only 41% of 406 customers surveyed in community and hospital pharmacies were interested in using electronic medicinal product information. Additionally, 54% of respondents indicated they would request a printed version from the pharmacy if the paper leaflet was not included in the package [11]. During the pandemic, a survey of 2518 vaccine recipients or their parents across 4 European countries (Belgium, Italy, Bulgaria, and France) reported an acceptance rate for electronic leaflets ranging from 55% to 82%, with an overall acceptability of 64% when a printed leaflet option was available [18].

Our patients’ perception of e-labeling as enhancing their understanding of medication aligns with findings from a Saudi Arabian study, where patients reported that reading medicinal product information leaflets positively impacted their knowledge about medicines and medication adherence [9]. However, only 1.6% (5/312) of participants in our study who obtained written medicine information used RiMUP, in contrast to the 91.1% utility rate of patient information leaflets observed in the Saudi Arabian study. This discrepancy may be attributed to the fact that RiMUPs are not distributed with products but are instead available as PDFs on the Malaysian NPRA website. HCPs are responsible for retrieving, printing, and disseminating them to patients if needed. Similar to experiences in Australia, this practice has not led to widespread dissemination of RiMUP as intended [27]. In our study, patients’ perceptions of the convenience of accessing e-labeling anytime and anywhere, as well as the ease of information retrieval, align with their primary source of medical information—the internet. Studies have shown that the availability of the internet has increased the use of online sources for medication information [11,18,28].

Malaysian patients have shown support for transitioning from paper medicinal product PIs to e-labeling for several reasons. First, there is widespread awareness of the negative impact of paper consumption associated with printing paper inserts [29]. This awareness is likely influenced by frequent media reports on extreme weather events and the broader effects of deforestation on climate change, which have heightened public concern about environmental issues. Second, the public adopted new health behaviors during the pandemic, which required transitioning many occupational and social activities to online platforms as a preventive measure against COVID-19 transmission [30]. Malaysians adapted to paperless systems such as QR codes and mobile apps, which explains the high preference for digital code scanning and medication apps among patients [31]. As socioeconomic activities resumed in the postpandemic period, this practice has become the new norm. By contrast, receiving a link to electronic medication information was the least favored option among patients. This reluctance may be attributed to the rising incidence of scams in Malaysia in recent years, which has made patients wary of clicking on links [32]. Additionally, several nationwide digitalization programs, such as the paperless road tax and online passport renewal policies recently introduced by the Malaysian government, have increased public acceptance of digital services [33]. This aligns with the mission of Malaysia’s National Fourth Industrial Revolution (4IR) policy, which aims to leverage digital technology to transform the economy in line with the Shared Prosperity Vision of creating a fair, equitable, and inclusive society by 2030 [34]. Additionally, the low perceived challenges related to digital gadget ownership, usage, and internet access may be due to ongoing income tax exemptions on laptops and the incentives promoting smartphone and laptop ownership. These measures have contributed to the public’s high readiness to adopt the e-labeling platform [35].

Paper leaflets for medicinal product information have an unavoidable environmental footprint, and shifting to electronic versions can significantly reduce production costs [12,16,28,36]. Additionally, features such as zooming and search functions on electronic devices make it easier and faster for patients to locate information [11,28,36,37]. These functionalities address the limitations of paper inserts and enhance the overall patient experience in managing their medication. This shift to e-labeling could potentially encourage patients who were previously hesitant to use medicinal product information leaflets to view electronic formats as a reliable source of information. Additionally, participants in our study suggested that the e-labeling system should present information in a comparative format across different drugs with the same indications and link to credible sources such as journals or research articles. This indicates that Malaysian patients are eager to learn about their medications and take an active role in managing their treatment. Providing patient-centric medicinal information in local languages can enhance medication literacy. Ultimately, e-labeling has the potential to improve medication use and lead to better health outcomes.

In our study, patients considered the legitimacy of the e-labels as a crucial aspect of the e-labeling system. Most patients preferred accessing e-labels through trusted platforms, such as government or official websites. This preference is likely due to the prevalent cybersecurity issues in Malaysia [38]. Consequently, patients emphasized the importance of maintaining system security to mitigate the risk of biased information that could impact patient safety. Our findings suggest that patients’ perceived limited skills in browsing e-labels correlate with lower acceptance of e-labeling, a phenomenon explained by Bandura’s Theory of Self-Efficacy [39]. According to this theory, individuals who feel confident in their ability to use the e-labeling platform are more likely to engage with and accept the technology. Consequently, providing a helpline for patients could facilitate their adaptation to the e-labeling system. Despite the generally high acceptance of e-labeling, 221/387 (57.1%) participants preferred not to completely eliminate paper inserts, a preference consistent with previous studies [11,18]. Therefore, it is important to implement procedures that support patients with limited digital skills until the e-labeling platform is fully established and effective.

Our study found that acceptance of e-labeling was lower among Chinese patients. Currently, the RiMUP is not available in Chinese or Tamil, the 2 major languages in our region of Malaysia, which may have impacted Chinese patients’ perceptions of e-labeling. Further research is needed to explore the underlying reasons for ethnic discrepancies in e-labeling acceptance among Malaysians. In our study, age was a significant factor in the univariate analysis but not in the multivariate analysis. This finding contrasts with Hammar et al [11], which suggested that older age might hinder the adoption of electronic patient information leaflets. The perceived lack of digital literacy skills among patients, which could be a more relevant factor, may have been reflected in our study, thereby minimizing age as a potential confounder. Similar results were reported in a recent European vaccine study that focused on individuals aged over 60 years [18].

### Implications for Policies and Strategies

Future strategies to enhance patient uptake of e-labeling should address concerns about the challenges associated with using digital platforms for medical information. First, flexibility should be provided to allow patients to request a printed copy of medicinal product information leaflets when necessary. Second, public awareness campaigns could encourage individuals who have not previously utilized medicinal product information leaflets to start using e-labeling, thereby increasing overall engagement. Patients should be informed about the benefits of e-labeling, including personalized information, enhanced medication safety, improved supply chain efficiency, and environmental protection. Educational materials should be provided in common local languages to ensure accessibility for patients from diverse backgrounds. Third, to help patients adapt to the e-labeling platform, practical demonstration videos with simple, clear instructions and visual aids can be displayed in pharmacy waiting areas to create a positive learning experience. Fourth, periodic reviews of the e-labeling system should be conducted to ensure it remains user-friendly and compatible with various electronic devices. Features such as linking e-labels to research studies and journals, enabling medication comparisons for the same indications, and including a section for public feedback can enhance the platform’s patient-centric approach. Fifth, a robust process should be established to update information in a centralized database. The responsible authority must verify and ensure the accuracy of the data before they are made available to the public. Lastly, a helpline should be provided to offer patients assistance whenever needed.

### Strength and Limitations

To the best of our knowledge, this is the first study assessing patient acceptance of e-labeling for medicinal product information during the postpandemic transition. The study also identified factors influencing patients’ acceptance of e-labeling. A stratified sampling method was used to ensure the sample accurately represents the patient population’s distribution in terms of age-related medication usage.

This study has several limitations. Conducted in a single hospital pharmacy located in Kuala Lumpur, a highly urbanized and densely populated city in Malaysia, the findings may not be generalizable to suburban or rural populations elsewhere in the country. Patients in rural areas may have limited internet access, which could influence their acceptance of e-labeling. Currently, the Malaysian government is working with the industry to enhance internet connectivity as part of the Madani Economy framework, aiming to provide stable and affordable internet access to Malaysians across all regions [40]. The proportion of participants with higher education levels in this study was greater than that observed in the national population census, which may introduce bias, as higher education is often linked with better economic status, ownership of digital devices, and proactive health information–seeking behavior [41]. However, the results from the multivariate analysis indicated that education level was not a significant predictor of e-labeling acceptance.

### Conclusions

Malaysian hospital patients demonstrated a moderately high level of acceptance of e-labeling of medicinal product information. Key factors predicting high acceptance included perceived benefits such as improved understanding of medication, environmental protection, and flexibility in information retrieval. By contrast, lower acceptance was associated with being of Chinese ethnicity and having perceived limitations in digital self-efficacy. Future strategies to enhance e-labeling uptake should focus on addressing patients’ concerns about digital platform challenges and emphasizing the advantages of e-labeling.

### Availability of Data and Materials

The data sets used and analyzed in this study are available from the corresponding author on reasonable request.

## Appendix

Multimedia Appendix 1 Questionnaire: Acceptance of electronic labeling among hospital ambulatory patients (English).

Multimedia Appendix 2 Questionnaire: Acceptance of electronic labeling among hospital ambulatory patients (Malay).

Multimedia Appendix 3 Questionnaire: Acceptance of electronic labeling among hospital ambulatory patients (Mandarin).

## Abbreviations

4IR: Fourth Industrial Revolution
AOR: adjusted odds ratio
CHERRIES: Checklist for Reporting Results of Internet E-Surveys
E-labeling: electronic labeling
E-labels: electronic labels
E-prescribing: electronic prescribing
FDA: Food and Drug Administration
HCP: health care professional
NPRA: National Pharmaceutical Regulatory Agency
PI: package insert
PMDA: Pharmaceuticals and Medical Devices Agency
RiMUP: Consumer Medication Information Leaflets
UMMC: University Malaya Medical Centre
UNSDG: United Nations Sustainable Development Goals
UTAUT2: Unified Theory of Acceptance and Use of Technology, second version
