# Supplementary material for: Acceptance of Electronic Labeling for Medicinal Product Information Among Malaysian Hospital Patients: Cross-Sectional Study
Source: J Med Internet Res. 2024 Sep 18;26:e56591. doi: 10.2196/56591 (PMC11447418; doi:10.2196/56591)
Supplement: Multimedia Appendix 1 [file jmir_v26i1e56591_app1.pdf]

Multimedia Appendix 1

Questionnaire: Acceptance to e-labelling among Hospital Ambulatory patients (English)

Section A: Demographic characteristics and use of pharmaceutical products package insert

1. **Age: __**

2. **Gender**
- Male
- Female

3. **Ethnicity**
- Malay
- Chinese
- Indian
- Others

4. **Highest level of education**
- University/ College
- Secondary School
- Primary School
- No formal schooling

5. **Do you ever obtain or seek written information about your medicine?**
- Yes
- No **(SKIP TO SECTION B)**

6. **What is your most common source of written information about medicine?**
- Medicinal product package insert
- Electronic Consumer Medication Information Leaflet (RiMUP) on NPRA website

- Internet (e.g Google)
- Leaflet from healthcare professionals (e.g. doctors, pharmacists, nurses)
- Others (please specify):__

7. Why did you choose the source that you selected in question 6? You may choose one or more options.

- Trustworthy
- Easy to understand
- Readily accessible
- Recommended by others
- Other reasons (please specify):__

8. How often do you read the accompanying medicinal product package insert?

- Always
- Sometimes
- Only when I receive a new medication
- Never

9. Please state the reason why you never read the accompanying medicinal product package insert? (SKIP TO SECTION B)

10. Why did you refer to the medicinal product package insert? You may choose one or more options.

- Medication purpose and how it works
- Dosage or administration
- Side effects
- Safety in pregnancy and breastfeeding
- Drug interactions or precaution with other diseases
- Others (please specify): __

Section B: Awareness of benefits and perceived challenges with electronic medicinal product information (5 Point Likert Scale)

Part I: The statements below are the potential benefits associated with electronic medicinal product information. For each statement, choose one answer that is applicable to you. (1= strongly disagree, 2= disagree, 3= neutral, 4= agree, 5= strongly agree).

No	Statement	Strongly disagree	Disagree	Neutral	Agree	Strongly agree
B1	Electronic Medicinal Product Information allows me to retrieve information anywhere, anytime, without fear of losing it.	1	2	3	4	5
B2	Electronic Medicinal Product Information allows me to understand my medication better with images and videos.	1	2	3	4	5
B3	Electronic Medicinal Product Information allows me to understand the medication better by choosing my preferred language.	1	2	3	4	5
B4	Electronic Medicinal Product Information allows the use of advanced interactive features such as adjustable font size keyword search and audio texts.	1	2	3	4	5
B5	Electronic Medicinal Product Information allows me to get the most updated medication information.	1	2	3	4	5
B6	Electronic Medicinal Product Information uses paperless system to protect the environment.	1	2	3	4	5

Part II: The statements below are the potential challenges with electronic medicinal products information implementation. For each statement, choose one answer that is applicable to you.. (Scale 1-5; where 1= strongly disagree, 2= disagree, 3= neutral, 4= agree, 5= strongly agree).

No	Statement	Strongly disagree	Disagree	Neutral	Agree	Strongly agree
BA1	I have no electronic gadgets to access electronic medicinal product information.	1	2	3	4	5
BA2	I have limited skill on the use of electronic gadgets to access electronic medicinal product information.	1	2	3	4	5
BA3	I have limited internet access and might face difficulties in using electronic medicinal product information.	1	2	3	4	5
BA4	I have limited skills to browse electronic medicinal product information.	1	2	3	4	5
BA5	I am concerned about obtaining potentially illegal electronic medicinal product information.	1	2	3	4	5

Section C: Acceptance and preference towards transition to electronic medicinal product information (5 Point Likert Scale)

The statements below are regarding the acceptance towards transition to electronic medicinal product information and preferences regarding its delivery**.** For each statement, circle one response that is applicable to you. (1= strongly disagree, 2= disagree, 3= neutral, 4= agree, 5= strongly agree).

No	Statement	Strongly disagree	Disagree	Neutral	Agree	Strongly agree
C1	I like the idea of substituting paper-based medicinal product package inserts with electronic medicinal product information in the near future.	1	2	3	4	5
C2	I would prefer to access electronic medicinal product information through scanning a digital code (Example: QR code) printed on the outer medication package.	1	2	3	4	5
C3	I would like to receive a link to the electronic medicinal product information sent through text message or an email.	1	2	3	4	5
C4	I would like to access the electronic medicinal product information through official or government websites.	1	2	3	4	5
C5	I would like to access the electronic medicinal product information through a digital patient service linked to my medication list. (Example: Medication Apps)	1	2	3	4	5
C6	I would like the option to request a printed copy of the medicinal product package insert.	1	2	3	4	5

C7. Do you have any other suggestions (e.g. support needs) towards the successful implementation of electronic medicinal product information?

- Yes, please elaborate:__________________
- No
