# Supplementary material for: Acceptance of Electronic Labeling for Medicinal Product Information Among Malaysian Hospital Patients: Cross-Sectional Study
Source: J Med Internet Res. 2024 Sep 18;26:e56591. doi: 10.2196/56591 (PMC11447418; doi:10.2196/56591)
Supplement: Multimedia Appendix 2 [file jmir_v26i1e56591_app2.pdf]

Multimedia Appendix 2

Questionnaire: Acceptance to e-labelling among Hospital Ambulatory patients (Malay)

Bahagian A: Ciri-ciri demografik dan penggunaan risalah bungkusan produk.

1. **Umur: __**

2. **Jantina**

- Lelaki
- Perempuan

3. **Bangsa**

- Melayu
- Cina
- India
- Lain-lain

4. **Tahap pendidikan tertinggi**

- Universiti/ Kolej
- Pendidikan menengah
- Pendidikan rendah
- Tidak bersekolah

5. **Pernahkah anda memperoleh atau mencari maklumat bertulis berkaitan ubat-ubatan anda?**

- Ya
- Tidak **(SKIP TO SECTION B)**

6. **Apakah sumber yang paling biasa anda gunakan untuk mencari maklumat bertulis tentang ubatan? Anda boleh memilih satu atau lebih jawapan.**
- Risalah bungkusan produk
- Risalah Maklumat Ubat Untuk Pengguna (RiMUP) elektronik di laman web Bahagian Regulatori Farmasi Negara (NPRA)
- Internet (Contoh: Google)
- Risalah dari Pakar kesihatan (Contoh: doktor, ahli farmasi, jururawat)
- Lain-lain (sila nyatakan): __

7. **Mengapakah anda memilih sumber yang telah anda pilih di soalan 6? Anda boleh memilih satu atau lebih jawapan.**
- Boleh dipercayai
- Senang difahami
- Senang diakses/diperoleh
- Disyorkan oleh orang lain
- Sebab lain (sila nyatakan): ___

8. **Berapa kerapkah anda membaca risalah bungkusan produk yang disertakan?**
- Sentiasa
- Kadang-kala
- Hanya apabila saya menerima ubatan yang baru
- Tidak pernah (Sila nyatakan sebab): __

9. **Nyatakan sebab anda tidak pernah membaca risalah produk ubat dalam bungkusan yang disertakan (SKIP TO SECTION B)**

10. **Mengapakah anda merujuk kepada risalah bungkusan produk? Anda boleh memilih satu atau lebih jawapan.**
- Tujuan ubatan dan cara ia berfungsi
- Dos atau cara penggunaan

- Kesan sampingan
- Keselamatan semasa mengandung dan menyusu
- Interaksi ubat atau langkah berhati-hati dengan penyakit lain
- Lain-lain (sila nyatakan): __

Bahagian B: Kesedaran tentang kebaikan dan cabaran dihadapi dengan Maklumat Produk elektronik (ePI) (Skala Likert 5 Mata)

Bahagian I: Kenyataan berikut adalah potensi faedah yang dikaitkan dengan ePI. Untuk setiap kenyataan, pilih satu jawapan yang berkenaan dengan anda. (1 = sangat tidak setuju, 2 = tidak setuju, 3 = neutral, 4 = setuju, 5 = sangat setuju).

No	Statement	Sangat tidak setuju	Tidak setuju	Neutral	Setuju	Sangat setuju
B1	Maklumat Produk Elektronik membenarkan saya memperoleh maklumat di mana-mana, pada pada bila-bila masa, tanpa risau tentang kehilangannya.	1	2	3	4	5
B2	Maklumat Produk Elektronik membenarkan saya memahami ubatan saya dengan lebih baik dengan menggunakan gambar dan video.	1	2	3	4	5
B3	Maklumat Produk Elektronik membenarkan saya lebih memahami tentang ubatan dengan memilih bahasa pilihan saya.	1	2	3	4	5
B4	Maklumat Produk Elektronik membenarkan penggunaan ciri interaktif yang canggih seperti saiz huruf yang boleh diubah, carian kata kunci dan audio teks.	1	2	3	4	5
B5	Maklumat Produk Elektronik membenarkan saya memperoleh maklumat tentang ubatan yang paling terkini.	1	2	3	4	5
B6	Maklumat Produk Elektronik menggunakan sistem tanpa kertas untuk melindungi alam sekitar.	1	2	3	4	5

Bahagian II: Kenyataan berikut adalah cabaran yang mungkin dihadapi dengan pelaksanaan ePI. Bagi setiap kenyataan, pilih satu jawapan yang berkaitan dengan anda. (1 = sangat tidak setuju, 2 = tidak setuju, 3 = neutral, 4 = setuju, 5 = sangat setuju)

No	Statement	Sangat tidak setuju	Tidak setuju	Neutral	Setuju	Sangat setuju
BA1	Saya tidak mempunyai alat elektronik untuk mengakses Maklumat Produk elektronik.	1	2	3	4	5
BA2	Saya mempunyai kemahiran yang terhad dalam penggunaan alat elektronik untuk mengakses Maklumat Produk elektronik.	1	2	3	4	5
BA3	Saya mempunyai akses internet yang terhad dan mungkin akan menghadapi kesukaran untuk menggunakan Maklumat Produk elektronik.	1	2	3	4	5
BA4	Saya mempunyai kemahiran yang terhad untuk melayari Maklumat Produk elektronik.	1	2	3	4	5
BA5	Saya tidak yakin tentang mendapatkan Maklumat Produk elektronik yang berpotensi menyalahi undang-undang.	1	2	3	4	5

Bahagian C: Penerimaan dan kecenderungan terhadap peralihan kepada Maklumat Produk elektronik (ePI)(Skala Likert 5 Mata)

Kenyataan berikut adalah tentang penerimaan terhadap peralihan kepada ePI dan kecenderungan kepada penyampaiannya. Bagi setiap kenyataan, sila pilih satu jawapan yang berkaitan dengan anda.

(1 = sangat tidak setuju, 2 = tidak setuju, 3 = neutral, 4 = setuju, 5 = sangat setuju)

No	Statement	Sangat tidak setuju	Tidak setuju	Neutral	Setuju	Sangat setuju
C1	Saya suka idea menggantikan risalah bungkusan dalam bentuk kertas dengan Maklumat Produk elektronik pada masa akan dat	1	2	3	4	5
C2	Saya lebih suka untuk mengakses Maklumat Produk elektronik dengan mengimbas kod digital (Contoh: Kod QR) yang dicetak di luar bungkusan ubat.	1	2	3	4	5
C3	Saya inginkan pautan untuk mengakses Maklumat Produk elektronik dihantar melalui mesej atau e-mel.	1	2	3	4	5
C4	Saya ingin mengakses Maklumat Produk elektronik melalui laman web rasmi atau laman web kerajaan..	1	2	3	4	5
C5	Saya ingin mengakses Maklumat Produk elektronik melalui perkhidmatan pesakit digital yang dikaitkan dengan senarai ubat saya. (Contoh: Aplikasi Ubat)	1	2	3	4	5
C6	Saya lebih suka jika diberi pilihan untuk meminta salinan bercetak risalah bungkusan.	1	2	3	4	5

C7. Adakah anda mempunyai cadangan lain (contohnya: keperluan sokongan) terhadap kejayaan pelaksanaan Maklumat Produk elektronik?

- Ya, sila huraikan:___________________
- Tidak
