# Supplementary material for: Acceptance of Electronic Labeling for Medicinal Product Information Among Malaysian Hospital Patients: Cross-Sectional Study
Source: J Med Internet Res. 2024 Sep 18;26:e56591. doi: 10.2196/56591 (PMC11447418; doi:10.2196/56591)
Supplement: Multimedia Appendix 3 [file jmir_v26i1e56591_app3.pdf]

Multimedia Appendix 3

Questionnaire: Acceptance to e-labelling among Hospital Ambulatory patients (Mandarin)

A ：个人基本资料和药品说明书的使用请选择最适合您的答案。

1. **年龄: __**

2. **性别**
- 男
- 女

3. **种族**
- 马来人
- 华人
- 印度人
- 其他

4. **最高学历**
- 大学 / 学院
- 中学
- 小学
- 没有受过正规教育

5. **您是否曾获取或征求有关您药物的书面信息?**
- 是
- 否 **(SKIP TO SECTION B)**

6. 以下哪个是你获得药物书面资讯的最主要途径？

- 药品说明书
- 国家药剂监管机构官网（NPRA）的电子消费药品说明书（RiMUP）
- 互联网（例如谷歌）
- 医务人员（例如医生、药剂师、护士）给予的传单
- 其他（请注明）：__

7. 为何您选择问题 6 的答案？您可以选择一个或多个选项

- 值得信赖
- 易于理解
- 易于获取
- 经他人推荐
- 其他原因（请说明）：__

8. 您多常阅读产品说明书?

- 经常
- 有时
- 只有当我收到新开的药物
- 从未（请说明原因）：__

9. 请说明从未阅读药品说明书的原因。 (SKIP TO SECTION B)

10. 您为什么会查询药品说明书？（您可以选择一个或多个选项）

- 用药目的及其作用
- 剂量或用法
- 副作用
- 怀孕与哺乳的安全性
- 药物的相互影响或与其他疾病需注意的事项

- 其他（请注明）：__

B. 了解电子药品信息的优势和挑战

第一部分：以下描述的是有关电子药品信息的潜在优势。请根据您对以下陈述的认同度，选择最合适您的答案。(1= 强烈反对，2=反对， 3=既不同意也不反对， 4= 同意， 5=非常同意)

No	Statement	强烈反对	反对	既不同意也不反对	同意	非常同意
B1	电子药品信息可以让我随时随地索取资讯，且不用担心会丢失它。	1	2	3	4	5
B2	电子药品信息可以让我通过图像和视频来更好地了解我的药物。	1	2	3	4	5
B3	电子药品信息可以让我通过选择我的首选语言来更好地了解我的药物。	1	2	3	4	5
B4	电子药品信息允许使用先进的互动功能，例如可调整字体大小，关键字搜索和语音功能。	1	2	3	4	5
B5	电子药品信息可以让我获得最新的药物信息。	1	2	3	4	5
B6	电子药品信息使用无纸化系统来保护环境。	1	2	3	4	5

第二部分：以下描述的是有关电子药品信息实施的潜在挑战。请根据您对以下陈述的认同度，选择合适的您的答案。

(1= 强烈反对，2=反对， 3=既不同意也不反对， 4= 同意， 5= 非常同意)

No	Statement	强烈反对	反对	既不同意也不反对	同意	非常同意
BA1	我没有电子产品来获取电子药品信息。	1	2	3	4	5
BA2	我在使用电子产品来获取电子药品信息方面的技能有限。	1	2	3	4	5
BA3	我的网络有限，在使用电子药品信息时可能会遇到困难。	1	2	3	4	5
BA4	我浏览电子药品信息的技能有限。	1	2	3	4	5
BA5	我对获取可能非法的电子药品信息感到不安	1	2	3	4	5

C. 电子药品信息的接受度和偏好

以下描述的是有关电子药品信息实施的接受度和对其传达方式的偏好。请根据您对以下陈述的认同度，选择最合适您的答案。（1= 强烈反对，2=反对， 3=既不同意也不反对，4= 同意， 5= 非常同意）

No	Statement	强烈反对	反对	既不同意也不反对	同意	非常同意
C1	我喜欢在不久的将来用电子药品信息代替纸质药品说明书的想法。	1	2	3	4	5
C2	我更倾向于通过扫描印在药物包装上的数字代码（例如：二维码）来获取电子药品信息。	1	2	3	4	5
C3	我想通过短信或电子邮件发送的链接获取电子药品信息	1	2	3	4	5
C4	我想通过官方或政府网站来查询电子药品信息。	1	2	3	4	5
C5	我想通过连接到我的药单的数码患者服务查询电子药品信息。（例如：药物应用程序）	1	2	3	4	5
C6	我想有选择性获取药品说明书的打印副本。	1	2	3	4	5

C7. 对于电子药品信息的成功实行，您有什么建议（例如需求）吗？

- 有，请详细说明:__________________
- 否
